# Aberrant alteration of follicular T helper cells in ulcerative colitis patients and its correlations with interleukin-21 and B cell subsets

**DOI:** 10.1097/MD.0000000000014757

**Published:** 2019-03-08

**Authors:** Guohui Xue, Yao Zhong, Lin Hua, Meijun Zhong, Xiaofeng Liu, Xueli Chen, Dian Gao, Nanjin Zhou

**Affiliations:** a Department of Clinical Laboratory, Jiujiang No. 1 People's Hospital, Jiujiang; b Department of Clinical Laboratory, The Second People's Hospital of Kunming, Kunming; c Jiangxi Academy of Medical Science, Nanchang; d Department of General Practice, The Second Affiliated Hospital of Nanchang University; e Department of Pathogen Biology and Immunology, Medical College of Nanchang University, Nanchang, China.

**Keywords:** biomarker, follicular T helper cells, interleukin-21, ulcerative colitis

## Abstract

Patients with ulcerative colitis (UC) are at increased risk of developing colitis-associated colon cancer. Accumulating evidence suggests that follicular T helper (T_FH_) cells play a crucial role in the pathogenic process of autoimmune diseases. However, little is known about the role of T_FH_ cells in the development of UC. To investigate the role of T_FH_ cells in the development of UC, the number of T_FH_ cells, the level of interleukin-21 (IL-21), the numbers of B cell subsets, and clinical parameters were detected in peripheral blood from 31 UC patients and 29 healthy controls. T_FH_ cells and the level of IL-21 were significantly higher in UC patients than in the healthy controls. A positive correlation between T_FH_ and IL-21 cells was found in UC patients. Moreover, aberrant frequencies of different subsets of B cells were observed in UC patients, and a positive correlation was found between CD38^+^CD19^+^ B cells and T_FH_ cells and between CD86^+^CD19^+^ B cells and T_FH_ cells. A high number of T_FH_ cells were positively associated with Mayo score, serum C-reaction protein (CRP) and serum IgG in UC patients. Our data indicate that T_FH_ cells and IL-21 are involved in the pathogenesis of UC.

## 1 Introduction

Ulcerative colitis (UC) is one of the major forms of inflammatory bowel diseases (IBDs) in humans. Patients with ulcerative colitis have an increased risk of developing colon cancer (CAC),^[1]^ and the risk rank is correlated with the duration and severity of the disease.^[2]^ Although the etiology and pathogenic process of UC remain unclarified, recent studies suggest that the abnormal interplay between T cells and other immune and nonimmune cells is pivotal in driving pathologic processes of UC,^[3,4]^ and T cell-derived cytokines are essential mediators of this cross-talk.^[5]^

The primary function of CD4^+^ T_H_ cells is to help the immune system fight infections. This function relies on the flexibility of naïve CD4^+^ T cells, which differentiate into diverse T_H_ cell subsets with specialized effector function by distinct pathogens.^[6]^ Under pathogenic conditions, aberrant levels of some cytokines promote the differentiation or function of specific effector T cell populations, which may unbalance the intestinal immune system and bring about a state of inflammation.^[3,4]^ Moreover, the differentiation of B cells to antibody-secreting cells requires the assistance of T_H_ cell subsets, and then, the production of neutralizing antibodies provides lasting protection against most pathogen infections. In this interplay, follicular T helper (T_FH_) cells play a key role in the development of antigen-specific B cells.^[7,8]^

T_FH_ cells are a specialized subset of CD4^+^ helper T cells located in germinal centres (GC) and are classically identified by their combined surface expression of inducible costimulator (ICOS), C-X-C chemokine receptor type 5 (CXC-R5), and programmed cell death-1 (PD-1), as well as the intracellular expression of the transcription factor Bcl-6.^[9–12]^ Notably, as shown recently, T_FH_ cells secrete IL-21 to modulate both humoural and cell-mediated immune responses in different murine and human autoimmune diseases, including systemic lupus erythaematosus (SLE), Sjögren's syndrome (SS), and rheumatoid arthritis (RA).^[13–15]^ However, little is known about how these different subsets of T_FH_ cells act in UC patients and whether T_FH_ cells are involved in the pathogenesis of UC.

IL-21 is a pleiotropic cytokine synthesized not only by T_FH_ cells but also by T_H_1 and T_H_17 cells in the germinal centres.^[14]^ IL-21 regulates the activation, proliferation, and survival of both CD4^+^ T cells and B cells, as well as the functional activity of CD8^+^ T cells and NK cells, and it limits the differentiation of inducible regulatory T cell.^[16]^ Clearly, IL-21 is involved in the regulation of central functions of the immune system. Elevated IL-21 has been observed in the gut of patients with IBD, especially in Crohn's disease (CD), which is thought to be caused by an exaggerated Th1 response against the luminal flora,^[5,17]^ whereas there are limited data about IL-21 in patients with UC.

In this context, to further investigate the potential relationship between the T_FH_ cells and clinical parameters of UC, we measured the proportions of circulating T_FH_ cells and activated B cells in patients with UC compared to those of healthy controls and analysed the correlation between the population ratio of T_FH_ cells and the IL-21 level, the proportion of B cell subsets, Mayo score, and other clinical-pathological features.

## 2 Method and materials

### 2.1 Patients

Patients gave informed consent for their samples to be analyzed in accordance with the Declaration of Helsinki. The study was approved by the Human Ethics Committee of Jiujiang First People's Hospital (Jiangxi, China). A total of 31 untreated UC patients and 29 sex- and age-matched healthy controls were recruited from our hospital. The diagnosis and Mayo score of 31 UC patients had been established by clinical, endoscopic, histological, and/or radiological criteria. Infection or the presence of parasites was excluded by stool culture and microscopic examination. The disease activity of active UC was determined using a grading scale including clinical and para-clinical variables. Another 29 subjects without prior history of chronic disease, autoimmune disease, cancer or recent infection were included as a healthy control group. Peripheral blood samples were collected from participants for in vitro cell culture and clinical analysis.

### 2.2 Blood sampling and analyses

Fasting venous blood samples were collected from individual healthy controls and UC patients. A portion of blood was used to isolate peripheral blood mononuclear cells (PBMCs) by density-gradient centrifugation using Ficoll-Paque PREMIUM (GE Healthcare Bio-science, Sweden), and the surplus blood samples were centrifuged for preparing to examine serum IL-21, IgA, IgM, and IgG.

### 2.3 Flowcytometry: surface antigen staining

PBMCs from UC patients and healthy controls were harvested and stained in duplicate for CD45-PC7, CD3-ECD, CD8-PE, CD4-FITC, CD19-PC5, CD38-FITC, CD27-PC7, CD86-PE (Beckman Coulter, Brea, CA), CXCR5-PC5 and ICOS-PE (eBioscience, San Diego, CA) at room temperature for 30 min. After being washed with PBS, the cells were characterized by flowcytometry analysis using the Beckman Coulter Cytomics FC500 (Beckman Coulter). Cells stained with separate antibodies were defined as certain T_FH_ cells (CD4^+^ CXCR5^+^ T_H_ cells and CD4^+^CXCR5^+^ICOS^+^ T_H_ cells) and subsets of B cells (CD38^+^CD19^+^ B cells, CD86^+^CD19^+^ B cells and CD27^+^CD19^+^ B cells). Data were analysed with a CXP Analysis Cytometer (Beckman Coulter).

### 2.4 IL-17A intracellular staining

Briefly, PBMC were resuspended in RPMI-1640 medium (Biological Industries, Kibbutz Beit Haemek, Israel) supplemented with 10% heat-inactivated foetal calf serum (Biological Industries) Then, the T_H_ cell subset was intracellularly stained for IL-17 after stimulation with phorbol 12-myristate 13-acetate (PMA) (50 ng/mL) and ionomycin (1 mg/ ml, Sigma-Aldrich) for 4 hours in the presence of brefeldin A (10 μg/mL BFA, BD Biosciences, San Jose, CA). Next, the T_H_ cell subset was incubated with the CD3-ECD and CD4-FITC antibodies. After fixation and permeabilization, cells were stained with PC5-conjugated anti-IL-17A (eBioscience).

### 2.5 Enzyme-linked immunosorbent assay (ELISA) for measurement of serum IL-21

The concentration of serum IL-21 in individual UC patients and HC was determined by ELISA using a human IL-21 ELISA kit (eBioscience) in accordance with the manufacturer's instructions. Individual serum at 1:4 dilutions were subjected to ELISA, and the concentrations of serum IL-21 in individual samples were calculated according to the standard curve established using the recombinant IL-21 provided.

### 2.6 Scattering nephlometry for measurement of serum IgA, IgM, and IgG

The concentrations of serum IgA, IgM, and IgG in individual UC patients and HC were examined by scattering nephlometry using an IMMAGE800 biochemical analyzer and matched reagents (Beckman Coulter) in accordance with the manufacturer's instructions.

### 2.7 Statistical analysis

Statistical analyses were performed by the GraphPad Prism 6.0 software (La Jolla, CA). The data are expressed as median and range unless specified. The difference between two groups was analysed by the Mann–Whitney *U* test. The relationship between variables was evaluated by Pearson's rank correlation test. A two-sided *P* value of <.05 was considered statistically significant.

## 3 Results

### 3.1 Patient characteristics

To assess the potential role of T_FH_ cells in the pathogenesis of UC, 31 Chinese patients with UC before treatment and 29 sex- and age-matched healthy controls (HCs) were recruited. Comparing the demographic and clinical characteristics of the groups, there was no significant difference in the concentration of serum IgA and IgM, or in the number of white blood cells (WBC) or lymphocytes (LYM), between these 2 groups. As expected, the concentrations of serum C-reactive protein (CRP) and IgG and the erythrocyte sedimentation rate (ESR) were significantly higher in the UC patients than those in the HCs (Table 1), suggesting that those patients were in a state of immune imbalance.

**Table 1:**
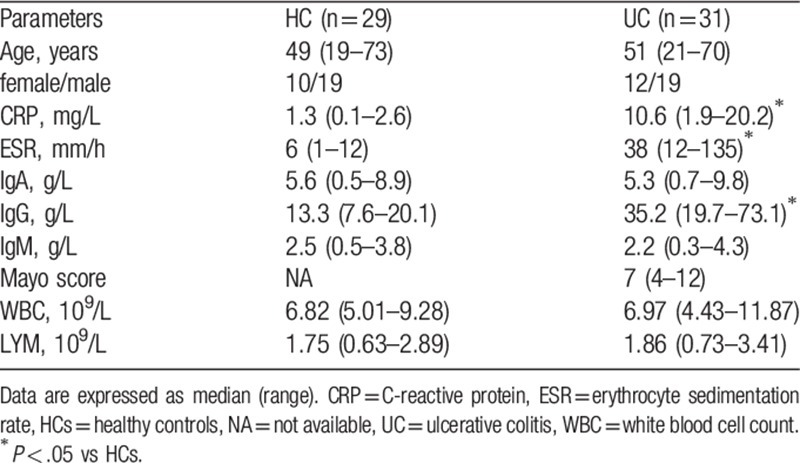
Demographic and clinical parameters of participants.

### 3.2 Increased proportions of circulating T_FH_ and T_H_17 cells in UC patients

**Figure 1:**
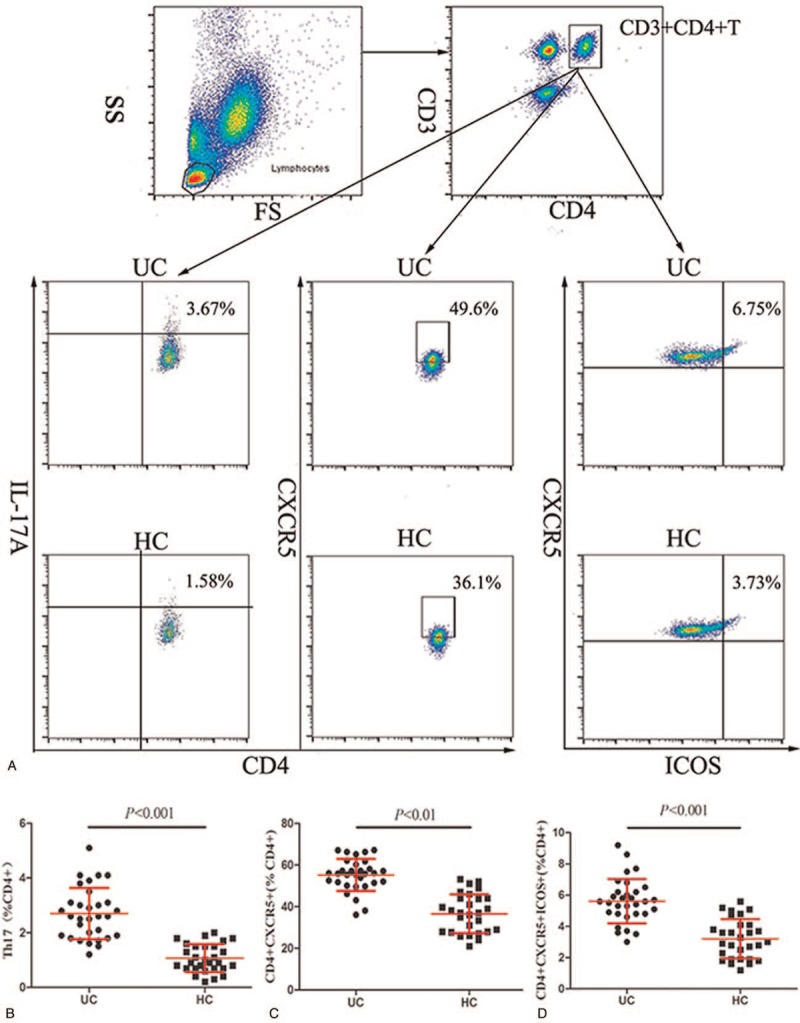
Flow cytometry analysis of T_FH_ and T_H_17 cells. (A) Flow cytometry analysis and (B–D) quantitative analysis. Data shown are representative dot plots or are expressed as the mean percentage of B cells of individual subjects. The difference between the two groups was analysed by the Mann–Whitney *U* nonparametric test. The horizontal lines represent the median values.

The numbers of T_FH_ cells and T_H_17 cells were analysed by flow cytometry (Fig. 1A). As shown in Figure 1B to D, the numbers of peripheral blood CD3^+^CD4^+^IL-17A^+^T_H_17 cells, CD3^+^CD4^+^CXCR5^+^T_FH_ cells and CD3^+^CD4^+^CXCR5^+^ICOS^+^ T_FH_ cells were significantly higher in the UC patients than those in the HCs (p<0.01, Fig. 1b-d). The higher numbers of CD4^+^CXCR5^+^ICOS^+^ T_FH_ cells and CD4^+^ IL-17A^+^ T_H_17 cells may contribute to the development of UC.

### 3.3 Elevated level of serum IL-21 is correlated to T_FH_ cells in UC patients

**Figure 2:**
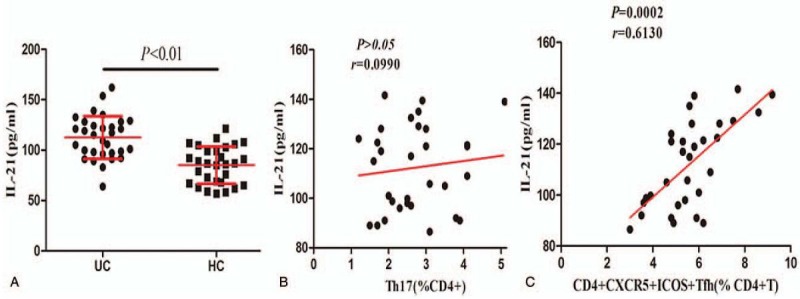
IL-21 is overexpressed in peripheral blood of UC patients, and its level correlates to T_FH_ cell level. (A) The serum IL-21 level was significantly elevated in UC patients compared to that in HCs. (B) No significant correlation between the level of IL-21 and the percentage of T_H_17 cells was found in UC patients. (**C**) Positive correlation between the level of IL-21 and the percentage of CD4^+^CXCR5^+^ICOS^+^ T_FH_ cells. UC = ulcerative colitis.

To explore the correlation between the level of IL-21 and the number of T cell subsets in UC, we detected their serum concentrations via ELISA, and correlation analysis was performed with Pearson's rank correlation. The level of IL-21 was significantly higher in UC patients than that in HCs (*P < *.01, Fig. 2A). There was no correlation between the level of IL-21 and the CD4^+^CXCR5^+^ T_FH_ cell proportion (data not shown). Interestingly, we observed a positive correlation between the level of IL-21 and the percentage of CD4^+^CXCR5^+^ICOS^+^ T_FH_ cells in UC patients (*P = *.0002, *r* = 0.6130, Fig. 2C) but no correlation between the level of IL-21 and the percentage of CD4^+^ IL-17A^+^ T_H_17 cells (*P > *.05, *r* = 0.0990, Fig. 2B). Our data indicate that IL-21 may be predominantly secreted by CD4^+^CXCR5^+^ICOS^+^ T_FH_ cells in UC patients, and this T_FH_ subset and IL-21 may participate in the development of UC.

### 3.4 Aberrant frequencies of different subsets of B cells in UC patients

**Figure 3:**
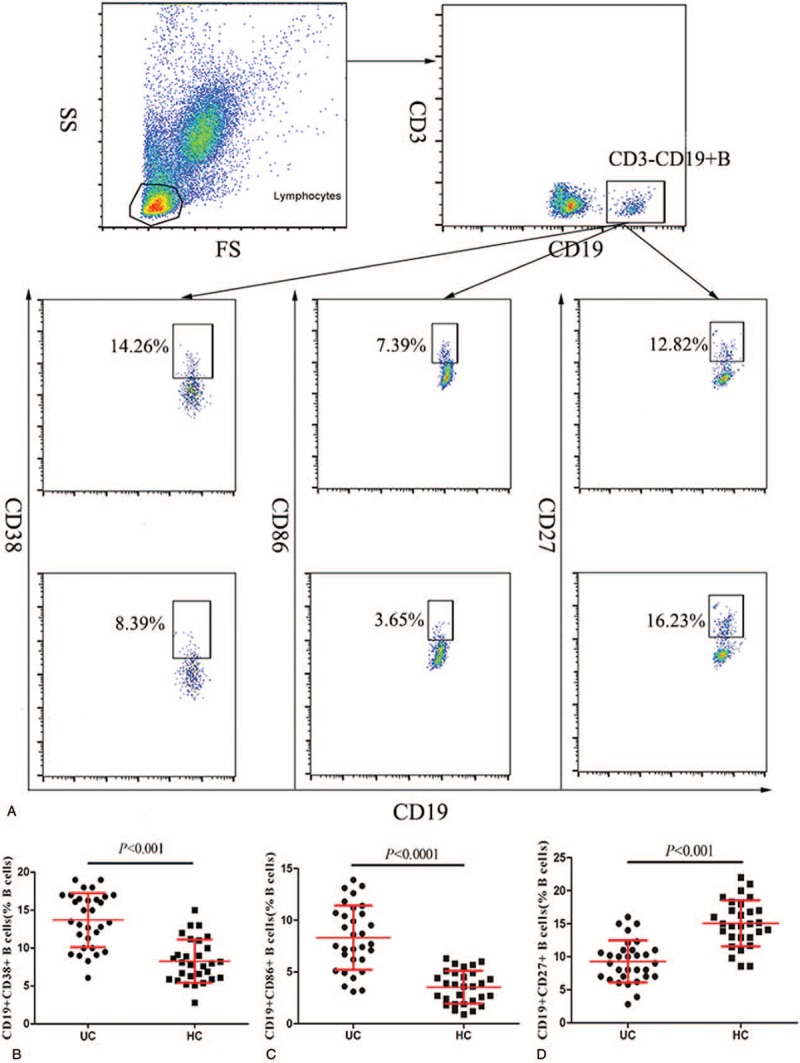
Flow cytometry analysis of B cell subsets. (A) Flow cytometry analysis and (B**–**D) quantitative analysis. Data shown are representative dot plots or are expressed as the mean percentage of B cells of individual subjects.

We next characterized the frequencies of different stages of B cells by flow cytometry (Fig. 3A) to assess the impact of B cells in the pathogenic process of UC. The percentages of CD38^+^CD19^+^ B cells and CD86^+^CD19^+^ B cells in the CD19^+^ B cell population of the UC patients were significantly higher than those in the HCs (*P < *.01, Fig. 3B and C). Interestingly, the percentage of CD27^+^CD19^+^ B cells in the UC patients was reduced compared to that in the healthy controls (*P < *.01, Fig. 3D). These data indicate that different subpopulations of B cells may contribute to the pathogenesis in UC patients.

### 3.5 The relationship between T_FH_ cells and B cell subsets in patients with UC

**Figure 4:**
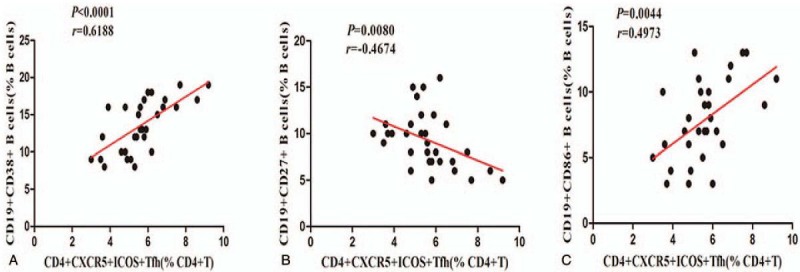
Correlation analysis between the percentages of T_FH_ cells and B cell subpopulations. (A and C) The percentages of CD38^+^CD19^+^B cells and CD86^+^CD19^+^B cells were positive correlated with CD4^+^CXCR5^+^ICOS^+^ T_FH_ cells. (B) Negative correlation between the percentages of CD27^+^CD19^+^ B cells and CD4^+^CXCR5^+^ICOS^+^T_FH_ cells.

T_FH_ cells can provide help for B cells by secreting cytokines and stimulating their surface molecules.^[7,9]^ We found that the percentage of CD38^+^CD19^+^ B cells in the CD19^+^ B cell population was positively correlated with the percentage of CD4^+^CXCR5^+^ICOS^+^ T_FH_ cells in the CD4^+^ T_H_ cell population of UC patients (*P < *.0001, *r* = 0.6188 Fig. 4A), and the percentage of CD86^+^CD19^+^ B cells was positively correlated with the percentage of CD4^+^CXCR5^+^ICOS^+^ T_FH_ cells in UC patients (*P = *.0044, *r* = 0.4973, Fig. 4C). The percentage of CD86^+^CD19^+^ B cells was negatively correlated with that of CD4^+^CXCR5^+^ICOS^+^ T_FH_ cells in UC patients (*P = *.0080, *r* = −0.4674, Fig. 4B).

### 3.6 The relationship between CD4^+^CXCR5^+^ICOS^+^ T_FH_ cells and clinicopathologic parameters of UC patients

**Figure 5:**
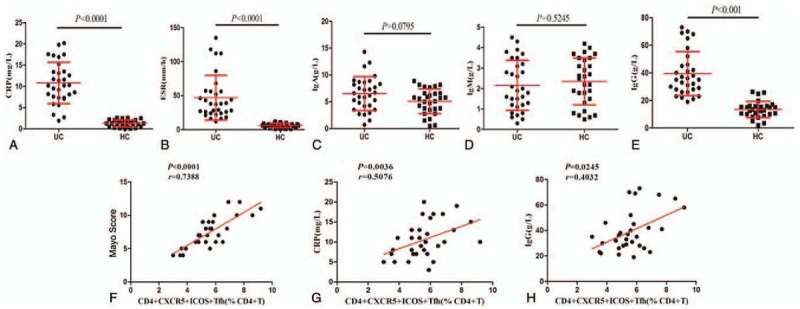
Comparison of clinical parameters in UC patients versus HCs. T_FH_ cell proportion was significantly correlated with Mayo score and the concentrations of CPR and IgG. (A–E) Quantitative analysis of clinical-pathological features. (F–H) Correlation analysis. The Mayo clinical score and the concentrations of serum CRP and IgG were positively correlated with the percentage of CD4^+^CXCR5^+^ICOS^+^ T_FH_ cells.

Having validated the relationship between T_FH_ cells and the clinical-pathological features of UC patients, we examined the correlations of abnormal clinical laboratory parameters with the number of T_FH_ cells in UC patients. As shown in Figure 5, the concentrations of serum CRP and IgG and the ESR of UC patients were significantly increased compared to those of the HCs (*P < *.0001, Fig. 5A, B, and E). No significant difference was found in the concentration of serum IgA (*P = *.0790, Fig. 5C) or IgM between groups (*P = *.5245, Fig. 5D). Importantly, compared to that of healthy controls, in patients with UC, the frequency of CD4^+^CXCR5^+^ICOS^+^ T_FH_ cells was significantly correlated with the Mayo score (*P < *.0001, *r* = 0.7388, Fig. 5F) and with the concentrations of serum CRP (*P = *.0036, *r* = 0.5076, Fig. 5G) and IgG (*P = *.0245, *r* = 0.4032, Fig. 5H). These results indicate that a change in the number of CD4^+^CXCR5^+^ICOS^+^ T_FH_ cells in UC patients may have potential as a biomarker to evaluate the severity and prognosis of the disease.

## 4 Discussion

Increasing evidence shows that the pathogenesis of IBD is associated with immunological abnormalities.^[4,5,18]^ T_FH_ cells, as a specific subset of CD4^+^ T helper cells, play an important role in immune balance.^[11]^ T_FH_ cells are involved in the pathogenesis of common autoimmune diseases;^[15]^ however, study about T_FH_ cells in UC disease is lacking. In the present study, we found that T_FH_ cells and T_H_17 cells were significantly more abundant in the peripheral blood of UC patients than in the HCs. The data suggest T_FH_ cells and T_H_17 cells may take part in the development of UC.

In addition, T_FH_ cells express high levels of CXCR5, which provides essential help for B cells to produce antibodies and for GCs to produce plasma and memory B cells.^[9,19]^ We found that levels of CD4^+^CXCR5^+^ T_FH_ cells were significantly elevated in the blood of UC patients, but there was no correlation with the level of IL-21. ICOS is expressed in activated T_FH_ cells, which stimulates the differentiation of activated T_FH_ cells through enhanced IL-21 expression and facilitates B cell antibody production.^[20]^ ICOS deletion leads to a profound decrease in memory B cell formation and to a switched antibody response in humans.^[21]^ Our data show that circulating CD4^+^CXCR5^+^ICOS^+^ T_FH_ cell and serum IgG levels were higher in UC patients than those in HCs, and a linear positive correlation existed between them, which indicated that ICOS may stimulate B cells to produce IgG to induce the development of UC.

IL-21 is produced mostly by activated CD4^+^ T cells and controls the differentiation and functional activity of effector T_H_ cells,^[14]^ whereas a high level of IL–21 triggers the inflammatory pathway and promotes tissue damage in some autoimmune diseases.^[22–25]^ A study by Ge et al^[26]^ reported that elevated intracellular IL-21 was correlated to the T_H_17 cell proportion in UC patients, suggesting that IL-21 is mainly produced by T_H_17 cells in UC. Nevertheless, our results demonstrate that the IL-21 level was significantly higher in the UC patients than in the HCs, and no correlation was found between serum IL-21 levels and T_H_17 cells in UC patients, while a significant positive correlation between IL-21 and CD4^+^CXCR5^+^ICOS^+^ T_FH_ cells was observed. Yu et al^[27]^ reported elevated levels of T_FH_ cells and IL-21 in tissues of UC patients and that IL-21-deficient mice were resistant to dextra sulfate sodium (DSS)-induced colitis and produced fewer T_FH_ cells. In combination with our data, these results indicate CD4^+^CXCR5^+^ICOS^+^ T_FH_ cells may be more closely related to the level of IL-21 than to other IL-21-synthesizing cells, and the secretion of IL-21 could be mainly through T_FH_ cells in UC patients. Certainly, in this regard, the main source of IL-21 in the UC pathogenic process necessitates further investigation, and more research is required to reveal the underlying mechanism of interaction between T_FH_ cells and IL-21 in UC.

A dysregulated frequency of B cell subsets has been implicated in UC,^[3,4]^ but the precise mechanism remains unclarified. There is general agreement that activated B cells highly express the CD86 costimulatory molecule, and CD86^+^ B cells can differentiate into CD38^+^ plasma B cells and then produce antibodies, such as IgM, IgG and IgA. Memory B cells express CD27 and can rapidly respond to antigens and generate immunoglobulin during secondary immune responses. In this study, the levels of circulating CD86^+^CD19^+^ activated B cells and CD38^+^CD19^+^ plasma B cells in UC patients were significantly higher than those in HCs, and levels of peripheral blood CD27^+^CD19^+^ memory B cells in UC patients were reduced compared to those of healthy controls. These data are consistent with previous studies,^[3,28]^ suggesting that inflammation may promote the development and redistribution of these three subsets of B cells. Alternatively, memory B cells may not play a major role in the pathogenesis of UC. Furthermore, through correlation analysis between T_FH_ cells and B cell subsets, we found that the proportions of activated B cells and plasm B cells were positively correlated that of T_FH_ cells and that the proportion of memory B cells was negatively correlated with that of T_FH_ cells. The basal level of IgG is highly dependent on T_H_ cells,^[29]^ and IgG is reduced markedly in the serum of ICOS^-/-^ mice.^[30]^ Similarly, we found that elevated serum IgG was correlated with the proportion of CD4^+^CXCR5^+^ICOS^+^ T_FH_ cells. These findings indicate that T_FH_ cells may represent a better biomarker to assess the dysregulation of B cell response in UC.

CRP is a nonspecific marker of systemic inflammation. A high level of CRP has been observed in IBD.^[31]^ We found that elevated CRP was positively correlated with elevated T_FH_ cells in UC patients, implying that the T_FH_ cells may have the potential to be a specific marker to estimate the inflammation degree of UC patients. Furthermore, the Mayo score, as a common disease activity index, provides a unified and standard assessment for the severity of UC, including stool frequency, rectal bleeding, mucosal appearance and physician rating of disease activity. However, measuring the Mayo score requires invasive examination and specialized calculation, which is subjective and slightly inaccurate. Our present study found that the Mayo score was well correlated with T_FH_ cell proportion, suggesting that T_FH_ cell percentage may have potential as a noninvasive biomarker to evaluate the activity of UC. Much more exploration is required to verify this hypothesis.

Collectively, our work demonstrates that circulating T_FH_ cells, activated B cells and plasma B cells may participate in the pathogenesis and development of UC, and IL-21 may be a crucial cytokine in the dysimmunity of UC. In UC, the level of IL-21 was strongly correlated with the level of T_FH_ cells, but not with T_H_17 cells. T_FH_ cells might become a novel noninvasive biomarker to assess immune state and the disease activity of UC. Further evidence on the mechanism and aetiology of UC await further study.

## Acknowledgments

We thank all participants in this study.

## Author contributions

Guohui Xue helped conceive the project. Yao Zhong and Guohui Xue performed the experiments, analysed the data and wrote the manuscript. Nanjin Zhou and Xueli Chen evaluated all the results and helped with the project. Lin Hua, Meijun Zhong, Xiaofeng Liu and Guohui Xue selected the patients, collected the samples and characterized the study cohort. Finally, all authors revised the manuscript. All authors obtained permission to acknowledge all those mentioned in the Acknowledgements.

**Conceptualization:** Nanjin Zhou, Dian Gao.

**Data curation:** Nanjin Zhou, Guohui Xue, Dian Gao.

**Formal analysis:** Yao Zhong, Lin Hua.

**Funding acquisition:** Guohui Xue, Lin Hua.

**Investigation:** Yao Zhong, Meijun Zhong, Xiaofeng Liu, Xueli Chen.

**Methodology:** Yao Zhong, Lin Hua, Xueli Chen.

**Project administration:** Nanjin Zhou, Guohui Xue.

**Resources:** Meijun Zhong, Xiaofeng Liu.

**Software:** Yao Zhong.

**Supervision:** Nanjin Zhou, Dian Gao.

**Validation:** Nanjin Zhou, Dian Gao.

**Visualization:** Nanjin Zhou, Dian Gao.

**Writing – original draft:** Guohui Xue, Yao Zhong.

**Writing – review & editing:** Guohui Xue, Yao Zhong, Meijun Zhong, Xiaofeng Liu, Xueli Chen.
